# Role of Biogenic Amines in Oviposition by the Diamondback Moth, *Plutella xylostella* L

**DOI:** 10.3389/fphys.2020.00475

**Published:** 2020-05-18

**Authors:** Fan Li, Ke Li, Li-Juan Wu, Yong-Liang Fan, Tong-Xian Liu

**Affiliations:** State Key Laboratory of Crop Stress Biology for Arid Areas and Key Laboratory of Integrated Pest Management on Crops in Northwestern Loess Plateau of Ministry of Agriculture, Northwest A&F University, Yangling, China

**Keywords:** biogenic amines, receptor, oviposition, regulation, insect reproduction

## Abstract

Oviposition is an important reproductive behavior that is triggered by mating in insects, and biogenic amines might be involved in its regulation. The effects of biogenic amines on oviposition have only been studied in a few insect species, and the findings to date have not been conclusive. In addition, there are few studies on the effects of biogenic amines on oviposition of the diamondback moth, *Plutella xylostella* L. Here, we tested how mating and biogenic amines regulate oviposition of *P. xylostella* by injecting amines and amine receptor antagonists into virgin and mated females and counting the number of eggs laid afterward. Biogenic amines of octopamine and tyramine could induce virgin adults of *P. xylostella* to lay eggs, while dopamine and serotonin had no such effect on oviposition. Furthermore, the octopamine antagonists mianserin, epinastine, and phentolamine inhibited oviposition by mated females. The tyramine antagonist yohimbine, dopamine antagonist SCH23390, and serotonin antagonist ketanserin did not block oviposition by mated females, and octopamine and tyramine-inducing oviposition by virgin females could be inhibited by the octopamine antagonists mianserin and epinastine instead of the tyramine antagonist yohimbine. We conclude that octopamine and its receptors are involved in mating-triggered oviposition in *P. xylostella*, while tyramine acts as a subsidiary. Further, the inducing effect of tyramine on oviposition is achieved via octopamine receptors instead of tyramine receptors. This experiment is helpful to further understand the role of biogenic amines in mating regulation and to provide a new strategy for controlling *P. xylostella*.

## Introduction

In insects, mating triggers many changes in the behavior and physiology of females, such as increasing oviposition, suppressing calling and pheromone synthesis, and re-mating (Gillott, 2003; Hirashima et al., 2007; Avila et al., 2011; Al-Wathiqui et al., 2016). Insect female oviposition can be elicited by some peptides and proteins transferred from male accessory glands through mating (Carmel et al., 2016) and by physical stimulation by males during mating (Giebultowicz et al., 1991). However, the underlining mechanisms and specific pathways in the downstream regulation of these factors are still unclear. In female moths, oviposition is usually activated within a few hours of mating (Kingan et al., 1995). Therefore, it is possible that a fast and sensitive neuropathic regulatory pathway participates in regulating insect oviposition.

Biogenic amines, including octopamine, tyramine, dopamine, and serotonin, serve as neurotransmitters, neuromodulators, and neurohormones, and they have essential effects on the regulation of physiological functions and behavior in insects (Roeder, 2005; Lange, 2009; Blenau and Thamm, 2011; Farooqui, 2012; Verlinden, 2018). As characteristic biogenic amines in invertebrates, octopamine and tyramine have similar functions as vertebrates adrenaline and norepinephrine, respectively (Blenau and Baumann, 2001). Octopamine is involved in a series of important behaviors and physiological activities, including olfactory learning and memory (Hammer and Menzel, 1998), rhythmic behaviors (Sombati and Hoyle, 1984), aggression (Zhou et al., 2008), feeding behaviors (Long and Murdock, 1983; Cohen et al., 2002), and energy metabolism (Mentel et al., 2003). Tyramine, which used to be regarded as the precursor of octopamine, also has neural functions in insects (Lange, 2009; Sinakevitch et al., 2017). Similar to octopamine in structure, tyramine shares most of its receptors with octopamine at different thresholds (Roeder, 2005; Farooqui, 2012). Besides some similar functions to octopamine, tyramine also has distinct effects on insect behavior such as regulating locomotion (Fox et al., 2006), excretion (Blumenthal, 2003; Cole et al., 2005), sensory signal processing (Blenau et al., 2000), gustatory responsiveness (Scheiner et al., 2017) and productive transition of queenless worker honeybees (Sasaki and Harano, 2007). Dopamine is widely distributed in vertebrates and invertebrates, and it has essential functions in learning and memory (Mizunami et al., 2009; Agarwal et al., 2011), motor behavior (Draper et al., 2007; Akasaka et al., 2010), sleeping and arousal (Kumar et al., 2012; Liu et al., 2012), and phase change (Ma et al., 2011) of insects. Serotonin (5-hydroxytryptamine, 5-HT) plays important roles in regulating rhythmic (Tomioka et al., 1993), aggression (Dierick and Greenspan, 2007), and the feeding behavior of insects (Dacks et al., 2003; French et al., 2014). In addition, biogenic amines are related to oviposition in different insects. Octopamine can induce oviposition in several insects such as the Indian meal moth, *Plodia interpunctella* (Rafaeli, 2004), and the rice leaf bug, *Trigonotylus caelestialium* (Yamane, 2014), but it inhibits oviposition of western tarnished plant bug, *Lygus hesperus* (Brent et al., 2016). Octopamine and tyramine were found to be essential in the regulation of *Drosophila* oviposition (Monastirioti, 2003; Cole et al., 2005). In two *Callosobruchus* species, tyramine can induce oviposition while octopamine has a limited inducing effect (Yamane and Miyatake, 2010). Besides octopamine and serotonin, dopamine and serotonin were also proved to be associated with oviposition in some species. For example, in virgin females of the fire ant, *Solenopsis invicta*, oviposition and oogenesis are associated with a high level of dopamine instead of octopamine (Boulay et al., 2001). In addition, dopamine was shown to induce egg-laying and maturing in the paper wasp, *Polistes chinensis*, whereas octopamine, tyramine, and serotonin had no such inducing effect (Sasaki et al., 2007). In contrast, serotonin prevented oviposition in the house fly, *Musca domestica* (Hays et al., 1969). However, the effects of biogenic amines on oviposition are not consistent in different insect species.

The diamondback moth, *Plutella xylostella* (Lepidoptera: Plutellidae), is a worldwide and destructive pest of cruciferous vegetables (Liu et al., 2008; Huang et al., 2018). This moth has the characteristics of a short life cycle, great fecundity, and notorious insecticide resistance (Gong et al., 2013). Research on the effects of biogenic amines on *P. xylostella* oviposition could provide insights into the regulation of biogenic amines on oviposition and new methods for the control of *P. xylostella*. However, there is no information on the relationship between biogenic amines and oviposition in *P. xylostella*.

With the purpose of understanding the effects of biogenic amines in regulating oviposition of *P. xylostella*, four biogenic amines were tested for their effects on virgin *P. xylostella* oviposition. Since gene silencing in Lepidoptera is hard to achieve (Guan et al., 2018), we used different amine receptor antagonists to explore the role of biogenic amines in mating-induced oviposition and determined the involvement of amine receptors in oviposition. Moreover, the blocking effects of different antagonists on biogenic amine-induced oviposition were also tested.

## Materials and Methods

### Study Insect

*Plutella xylostella* larvae were fed on cabbage plants (*Brassica oleracea* L. var. Qingan 70) at 25 ± 1°C, 60% RH, and 16L: 8D conditions. Pupae were transferred individually into 2 ml micro-centrifugal tubes with air holes on the surface until they emerged. The newly emerged adults were picked up every day and labeled as 1-day-old. Adults were screened based on their weights (females: 4.23 ± 0.70 mg; males: 2.74 ± 0.35 mg). Adults with body weights greater than these standards were deemed eligible for experiments. Adults were fed individually with a 10% sucrose solution (Huada, Shantou, Guangdong, China).

### Mating Arrangements

A 2-day-old virgin female and a 2-day-old virgin adult male were put together into a cylindrical transparent box (diameter: 9 cm; height: 12 cm) 1 h before scotophase. Copulation was checked every 30 min during scotophase with a dim red light. Females that kept coupling for more than 30 min and finished coupling naturally were regarded as mated. In addition, a 2-day-old virgin female was put into a box alone as the control.

### Injection of Biogenic Amines Into Virgin Females

Octopamine hydrochloride (Sigma-Aldrich, Louis, MO, United States), tyramine hydrochloride (Sigma-Aldrich, Louis, MO, United States), dopamine hydrochloride (Sigma-Aldrich, Louis, MO, United States), and serotonin hydrochloride (Sigma-Aldrich, Louis, MO, United States) were dissolved in Milli-Q water at concentrations of 0.0001, 0.001, 0.01, 0.1, and 0.2 mol/L. Two-day-old virgin females were narcotized with CO_2_. Volumes of 0.3 μL of the different amine solutions at different concentrations were injected slowly into females through the intersegmental membrane between the 4th and 5th segments, using the Nanoinject II micro-injection instrument (Drummond Scientific, Broomall, PA, United States). The preparation of capillary needles were performed according to the methods mentioned by Chen et al. (2016). As the control, 0.3 μL of Milli-Q water was injected into virgin females.

### Injection of Amine Receptor Antagonists Into Mated Females

Mianserin hydrochloride (Aladdin, Los Angeles, CA, United States), epinastine hydrochloride (Aladdin, Los Angeles, CA, United States), phentolamine hydrochloride (Aladdin, Los Angeles, CA, United States), yohimbine hydrochloride (Aladdin, Los Angeles, CA, United States), SCH23390 hydrochloride (Aladdin, Los Angeles, CA, United States), and ketanserin hydrochloride (Aladdin, Los Angeles, CA, United States) were dissolved into Milli-Q water at the concentrations of 0.005and 0.02 mol/L. Two-day-old virgin females and males were mated using the aforementioned method. Mated females were injected with 0.3 μL of each of six different amine receptor antagonists at different concentrations. As the control, 0.3 μL of Milli-Q water was injected into mated females using the method mentioned above.

### Injection of Antagonists Mixed With Octopamine or Tyramine

Octopamine hydrochloride or tyramine hydrochloride and each of the different antagonists (i.e., mianserin hydrochloride, epinastine hydrochloride, phentolamine hydrochloride, yohimbine hydrochloride, SCH23390 hydrochloride, or ketanserin hydrochloride) were dissolved into Milli-Q water to get different solutions containing 0.1 mol/L octopamine or tyramine and 0.02 mol/L antagonist. Two-day-old virgin females were narcotized with CO_2_ and injected with 0.3 μL of different mixed solutions using the same method mentioned above. As the control, 0.3 μL of Milli-Q water was injected into virgin females.

### Egg Counting

Each female from the treatment and control groups was transferred into a 3 cm petri dish containing fresh cabbage leaves and fed with a 10% source solution. Counting number of eggs and renewal of cabbage leaves and source solution in petri dishes were executed in every 24 h for 5 days.

### Detection of Biogenic Amines Using High Performance Liquid Chromatography–Mass Spectrometry (HPLC–MS)

The methods of HPLC was designed according to the methods described by Ma et al. (2015), which was used for detection of octopamine and tyramine in locust. Two-day-old mated female and virgin female adults were collected, respectively. Mated females were collected 1 h after mating. Wings-off females in a group of three per sample were ground in a 1.5 mL micro-centrifuge tube. After grinding, sample was homogenized in 100 μL of 0.1 mol/L perchloric acid for 10 min. Then homogenized sample was centrifuged using a table-top micro-centrifuge (Eppendorf International, Hamburg, Germany) at 14,000 g for 10 min at 4°C. The supernatant was transferred into a chromatographic sample bottle and stored at −80°C for analysis. HPLC-MS (Thermo Scientific, Waltham, MA, United States) was equipped with C18 chromatographic column (Intertsil OSD-4 C18, 250mm × 3.0mm, 5 μm, Shimadzu Corporation). The flow rate was set at 250 μL/min with a temperature at 5°C. The mobile phase was composed of 7% acetonitrile (Sigma-Aldrich, Louis, MO, United States), 90 mmol/L monobasic phosphate sodium (Huada, Shantou, Guangdong, China), 50 mmol/L citric acid (Sigma-Aldrich, Louis, MO, United States), 2 mmol/L octanesulfonic acid (Sigma-Aldrich, Louis, MO, United States), 2 mmol/L NaCl (Huada, Shantou, Guangdong, China), and 50 mmol/L EDTA (Sigma-Aldrich, Louis, MO, United States). Then the LTQ XL linear ion trap mass spectrometer (Thermo Scientific, Waltham, MA, United States) with electrospray ionization source (ESI) was employed for analysis of separated samples. Target components were identified by scan and fragmentation using data dependent MS/MS as well as by comparing with four biogenic amine standards. Data were processed by Xcalibur 2.1 software (Thermo Scientific, Waltham, MA, United States).

### Statistical Analysis

All data are expressed as the means ± standard error (SE). The Statistical Package for the Social Sciences 19.0 software (SPSS Inc., Chicago, IL, United States) was employed for statistical analyses. Significant differences between samples were determined using a Student’s *t*-test and one-way analysis of variance (ANOVA), Duncan and Tamhane’s T2 test at 5 and 0.1% significance levels.

## Results

### Role of Mating in Regulating Oviposition by *P. xylostella* Females

**FIGURE 1:**
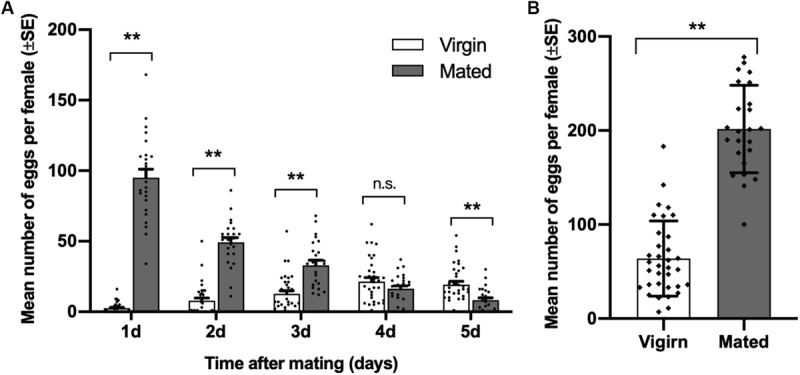
Effect of mating on female oviposition by *Plutella xylostella*. **(A)** Number of eggs laid by virgins (*n* = 34) and mated females (*n* = 24) one to 5 days after mating. **(B)** Total number of eggs laid by virgins (*n* = 34) and mated females (*n* = 24) for 5 days after mating. Individual data is displayed with dots. **indicates a significant difference (*p* < 0.01, Student’s *t*-test).

Compared with virgin females of diamondback moth, the number of eggs laid by mated females was significantly higher on days 1 to 3 after mating, and it was highest on the first day after mating. On day 5 after mating, the number of eggs laid by mated females was lower than that of virgin females (day 1, *t* = 15.025, df = 23.491, *p* < 0.001; day 2, *t* = 10.771, df = 37.468, *p* < 0.001; day 3, *t* = 4.751, df = 39.820, *p* < 0.001; day 4, *t* = −1.366, df = 56, *p* = 0.177; day 5, *t* = −3.870, df = 55.560, *p* = 0.001; Figure 1A). Meanwhile, the total number of eggs laid by mated females in 5 days was significantly higher than virgin females (*t* = 11.767, df = 44.780, *p* < 0.001; Figure 1B).

### Effects of Biogenic Amines on Oviposition by Virgin *P. xylostella* Females

**FIGURE 2:**
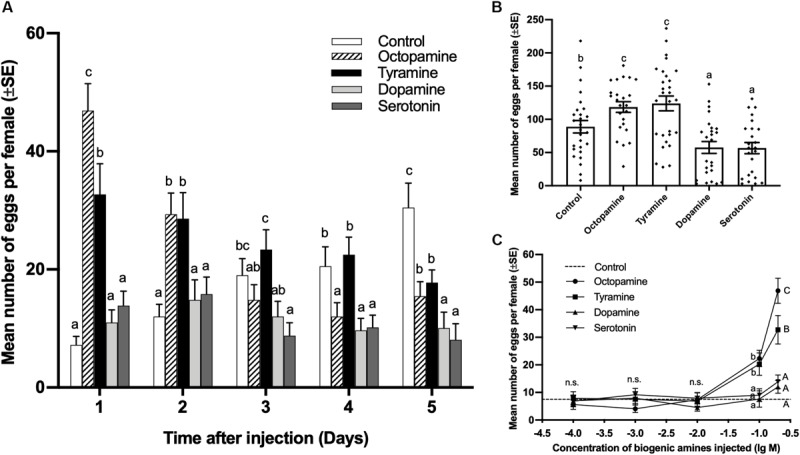
Effect of biogenic amine injections on oviposition by virgin females of *Plutella xylostella*. Number of eggs laid by females from days 1–5 **(A)**, and total number of eggs laid by females in 5 days **(B)** after injection with 0.3 μL of 0.2 mol/L octopamine (*n* = 24), tyramine (*n* = 30), dopamine (*n* = 25), serotonin (*n* = 24), and Milli-Q water as a control (*n* = 27). Individual data is displayed with dots. **(C)** Dose-response study of the effects of biogenic amines on the number of eggs laid by virgin females within 24 h after injection (24 ≤ *n* ≤ 32). Different letters on the top of bars indicate significant differences between treatments on the same day (*p* < 0.05, ANOVA, Duncan). The dotted line in **(C)** indicates the number of eggs laid by virgin females injected with Milli-Q water.

The number of eggs laid by virgin females injected with 0.3 μL of 0.2 mol/L octopamine and tyramine was significantly higher than the control on days 1 and 2, while the dopamine and serotonin treatments showed no significant difference to the control on days 1 and 2 after injection. Although both octopamine and tyramine could induce virgin females to lay eggs, the females injected with octopamine laid more eggs than tyramine treatments (day 1, df = 4,125, *F* = 22.496, *p* < 0.001; day 2, df = 4,125, *F* = 5.720, *p* < 0.001; Figure 2A). On day 3, the females injected with serotonin laid less eggs than control, while there was no significant difference among the control and other amine treatments (df = 4,125, *F* = 4.371, *p* = 0.002; Figure 2A). On day 4, except for the tyramine treatment, the number of eggs laid in each of other biogenic amine treatments was significantly lower than in the control (df = 4,125, *F* = 5.380, *p* < 0.001; Figure 2A). On day 5, the number of eggs laid in each of other biogenic amine treatments was significantly lower than in the control (df = 4,125, *F* = 10.067, *p* < 0.001; Figure 2A).

The total number of eggs laid by females injected with 0.2 mol/L octopamine and tyramine within 5 days after injection was significantly higher than that for the control, while dopamine and serotonin treatments were significantly lower than the control (df = 4,123, *F* = 11.669, *p* < 0.001; Figure 2B).

A gradient of doses of biogenic amines were injected into virgin females, and the number of eggs was counted 24 h after injection. There was no significant difference between contorl and biogenic amine tratments at concentrations of 0.0001, 0.001, and 0.01 mol/L. At concentrations of 0.1 and 0.2 mol/L, the number of eggs laid by virgin females injected with octopamine and tyramine was significantly higher than for the control, while there were still no significant difference between dopamine and serotonin treatments and control. The number of eggs laid by females injected with 0.2 mol/L of octopamine was significantly more than that for the tyramine treatment (0.0001 mol/L, df = 4,118, *F* = 0.172, *p* = 0.952; 0.001 mol/L, df = 4,118, *F* = 1.025, *p* = 0.398; 0.01 mol/L, df = 4,119, *F* = 0.492, *p* = 0.741; 0.1 mol/L, df = 4,133, *F* = 10.773, *p* < 0.001; 0.2 mol/L, df = 4,125, *F* = 20.909, *p* < 0.001; Figure 2C).

### Effects of Different Biogenic Amine Receptor Antagonists on Mating-Induced Oviposition

**FIGURE 3:**
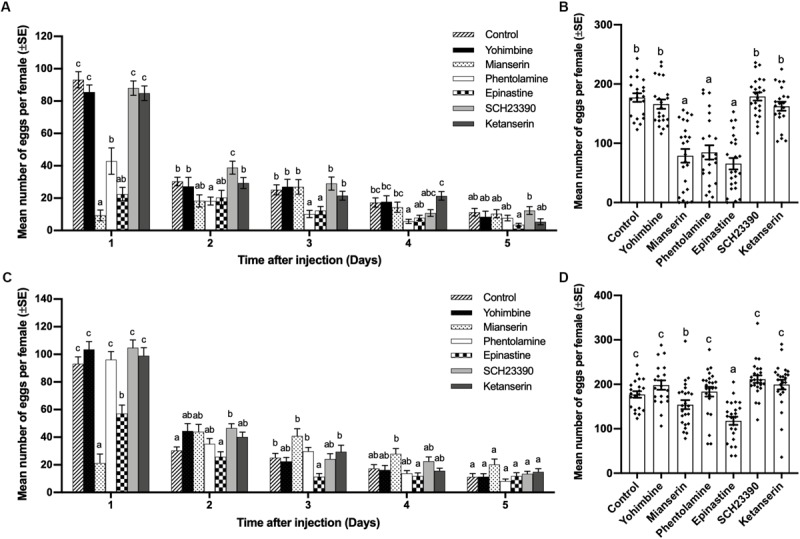
Effect of different biogenic amine antagonists on mating-induced oviposition by *Plutella xylostella*. Number of eggs laid by mated females from days 1–5 after injection with 0.3 μL of 0.02 mol/L **(A)** or 0.005 mol/L **(C)** antagonists and Milli-Q water as a control (20 ≤ *n* ≤ 24). Total number of eggs laid by mated females for 5 days after injection with 0.3 μL of 0.02 mol/L **(B)**, or 0.005 mol/L **(D)** antagonists and Milli-Q water as a control (20 ≤ *n* ≤ 24). Individual data is displayed with dots. Different letters on the top of bars in **(A–D)** indicate significant differences between treatments on the same day (*p* < 0.05, ANOVA and Tamhane’s T2 test).

At a concentration of 0.02 mol/L, mianserin, epinastine, and phentolamine showed the ability to inhibit oviposition by mated females. Mianserin had the strongest but shorter inhibiting effect for 1 day, while phentolamine and epinastine had longer inhibition durations of 4 and 3 days, respectively. Yohimbine, SCH23390, and ketanserin had no significant influence on mated female oviposition one to 5 days after injection (day 1, df = 6,152, *F* = 47.611, *p* < 0.001; day 2, df = 6,152, *F* = 4.108, *p* = 0.001; day 3, df = 6,152, *F* = 4.908, *p* < 0.001; day 4, df = 6,152, *F* = 5.064, *p* < 0.001; day 5, df = 6,152, *F* = 1.974, *p* = 0.073; Figure 3A). The total number of eggs laid by mated females over 5 days was also significantly decreased by mianserin, phentiolamine, and epinastine, while other antagonists had no significant effect (df = 6,152, *F* = 30.618, *p* < 0.001; Figure 3C). At a concentration of 0.005 mol/L, mianserin and epinastine reduced the number of eggs laid by mated females, and the reducing effects lasted for 1 and 3 days, respectively. On the first day after injection, 0.005 mol/L of mianserin had a stronger inhibiting effect than epinastine (day 1, df = 6,154, *F* = 29.472, *p* < 0.001; day 2, df = 6,154, *F* = 3.801, *p* = 0.001; day 3, df = 6,154, *F* = 5.766, p < 0.001; day 4, df = 6,154, *F* = 3.994, *p* = 0.001; day 5, df = 6,154, *F* = 2.338, *p* = 0.035; Figure 3B). Mianserin and epinastine both decreased the total number of eggs laid by mated females over 5 days, while epinastine had a stronger inhibiting effect than mianserin (df = 6, 154, *F* = 11.271, *p* < 0.001; Figure 3D). A concentration of 0.005 mol/L of yohimbine, phentolamine, SCH23390, and ketanserin had no significant influence on the number of eggs laid by mated females one to 5 days after injection (Figures 3B,D).

### Effects of Biogenic Amine Receptor Antagonists on Octopamine and Tyramine-Induced Oviposition

**FIGURE 4:**
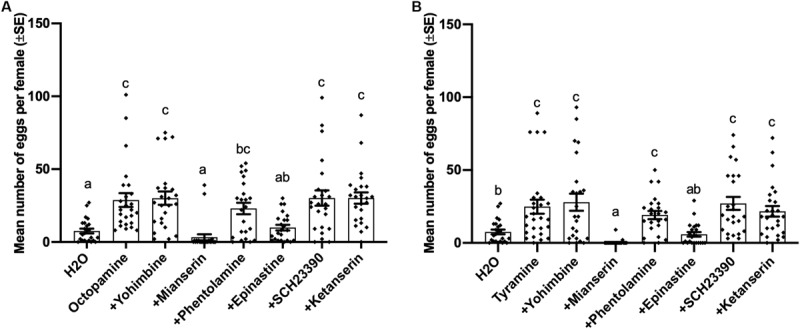
Effects of different antagonists on octopamine-inducing and tyramine-inducing oviposition by virgin females of *Plutella xylostella*. Number of eggs laid by virgin females at 1 day after injection with 0.3 μL of different mixed solutions containing 0.02 mol/L different antagonists and 0.1 mol/L octopamine **(A)** or tyramine **(B)** and Milli-Q water as a control (23 ≤ *n* ≤ 27). Individual data is displayed with dots. Different letters above columns mean significant differences between treatments (*p* < 0.05, ANOVA and Tamhane’s T2).

Oviposition induced by both octopamine (df = 7, 183, *F* = 9.463, *p* < 0.001) and tyramine (df = 7,185, *F* = 8.334, *p* < 0.001) was also suppressed by 0.02 mol/L mianserin and epinastine, while other antagonists failed to block the inducing effect of tyramine and octopamine on virgin female oviposition (Figures 4A,B).

### Detection of Biogenic Amines in Female Adults of *P. xylostella*

With HPLC-MS method we failed to detect any of the biogenic amines in either virgin or mated females.

## Discussion

The main findings of this study on *P. xylostella* were that mating-induced oviposition; octopamine and tyramine induced virgin females to lay eggs whereas dopamine and serotonin slightly inhibited oviposition; mianserin, phentiolamine, and epinastine inhibited egg-laying of mated females whereas yohimbine, SCH23390, and ketanserin did not; and mianserin and epinastine blocked octopamine and tyramine-induced oviposition in *P. xylostella*. These findings are discussed below.

In *P. xylostella*, mating was found to induce oviposition behavior for a few days (Figure 1), and oviposition commenced immediately after finishing mating, which indicates that oviposition by the diamondback moth might be supported by the nervous system. Biogenic amines, which are neurotransmitters, neuromodulators, and neurohormones (Monastirioti, 1999; Blenau and Baumann, 2001), have important effects on post-mating behaviors in insects, including increasing the number of eggs laid (Hirashima et al., 2007; Yamane and Miyatake, 2010; Wu et al., 2017). However, the effects of biogenic amines on oviposition by insects are unstable and inconsistent with each other in different species (Boulay et al., 2001; Monastirioti, 2003; Rafaeli, 2004; Cole et al., 2005; Yamane and Miyatake, 2010; Brent et al., 2016).

The finding that exogenous tyramine and octopamine induced oviposition by the virgin females of *P. xylostella* (Figure 2) is consistent with findings from some studies. Rafaeli (2004) found that octopamine and its two analogs could induce oviposition by virgin females in a similar species, *P. interpunctella*, and octopamine and tyramine were both found to be essential for female oviposition in *Drosophila melanogaster* (Monastirioti, 2003; Cole et al., 2005). When lacking octopamine, *Drosophila* females failed to release mature eggs from the ovary (Monastirioti, 2003). Interestingly, *Drosophila* females lacking tyramine could release the mature oocytes from the ovary into the oviduct but could not deposit the eggs normally. Moreover, extrinsic octopamine had a stronger rescue effect than tyramine on the sterility of a *Drosophila* tyramine mutant (Cole et al., 2005). This phenomenon is in accordance with our results that tyramine had a weaker inducing effect on *P. xylostella* oviposition than octopamine (Figures 2A,C). This means tyramine might only act as an assistant and synthetic source of octopamine and have partial effects of octopamine in insect oviposition. The significantly low total number of eggs laid for 5 days in the dopamine and serotonin treatments (Figure 2B) indicate that dopamine and serotonin might have slightly inhibiting effects on the oviposition by *P. xylostella*. This is similar to findings for the house fly *M. domestica* and the locusts *Locusta migratoria*, in which serotonin inhibited oviposition and oviduct relaxation (Hays et al., 1969; Lange, 2004). However, in the fire ant *S. invicta* and the wasp *P. chinensis*, dopamine induced the oviposition and development of reproductive systems (Boulay et al., 2001; Sasaki et al., 2007). As effects similar to those of dopamine are not detected in any species other than social insects, dopamine might indeed have an inducing effect on oviposition only in social insects. Besides, the HPLC-MS was employed to detect the changes of biogenic amines in *P. xylostella*. However, none of the biogenic amines was detected in our experiments, which means the concentration of biogenic amines in whole bodies of *P. xylostella* might be below the threshold of detection method described above. Octopamine could be produced at specific region and transferred from abdomen nerve terminal to reproductive tracts (Monastirioti, 2003). Hence it might be hard to detect octopamine from whole bodies or haemolymph of small insects like the diamondback moth. The detection of biogenic amines using HPLC mainly occurred in large insects such as locust and crickets (Nagao and Tanimura, 1988; Ma et al., 2015). More different attempts needed to be made for detecting of biogenic amines in *P. xylostella.* In *Drosophila*, mating increased the number of octopaminergic type II boutons around the lateral and common oviducts (Rodríguez-Valentín et al., 2006; Kapelnikov et al., 2008), which can prove that mating induces the production and release of octopamine. Unlike endogenesis biogenic amines which could be transferred from nerve terminal to specific action sites (Monastirioti, 2003), the injected biogenic amines will be diluted in haemolymph and cannot be transferred into the specific region accurately. Therefore, a higher concentration of exogenous biogenic amines might be needed for imitating the effects of endogenesis biogenic amines. As the biosynthetic intermediate precursor for octopamine, tyramine is similar to octopamine in structure (Lange, 2009) and shares receptors and partial functions with octopamine (Donini and Lange, 2004). Based on structural similarities with vertebrate adrenergic receptors, invertebrate octopaminergic receptors have been divided into α-adrenergic-like receptors (Octα-R) and β-adrenergic-like receptors (Octβ-Rs, including Octβ1-R, Octβ2-R, and Octβ3-R). The invertebrate tyraminergic receptors (TYR-R) have been divided into TYR1-R, which used to be the octopaminergic/tyraminergic receptor, and TYR2-R (Evans and Maqueira, 2005; Farooqui, 2012). Except for TYR1-R and TYR2-R, which are the original targets of tyramine, the octopamine original receptors Octα-R and Octβ-R can also be activated by tyramine in an appropriate concentration. The active Octα-R has a main effect of increasing the concentration of intracellular Ca^2+^ (calcium), and it can also increase cAMP (cyclic adenosine monophosphate) levels slightly with an extremely high threshold (Balfanz et al., 2005; Ohtani et al., 2006). Octβ-R can specifically increase the intracellular cAMP level (Farooqui, 2012; Kita et al., 2016). Although they can bind with tyramine and get activated, both Octα-R and Octβ-R have a higher affinity for octopamine than tyramine (Farooqui, 2012). TYR1-R has a function of decreasing intracellular cAMP levels when coupling with tyramine, and it has a weaker decreasing effect when coupling with octopamine (Blenau et al., 2000; Poels et al., 2002). In addition, TYR1-R also increases intracellular Ca^2+^ when coupling with tyramine and octopamine while TYR2-R is only combined with tyramine (Huang et al., 2009). Therefore, it is still not clear which type of amine receptors is involved in the regulation of oviposition, although octopamine and tyramine can imitate mating-induced oviposition. As the efficiency of RNA interference in Lepidoptera is low, different amine receptor antagonists were used in this experiment to corroborate the roles of amines in mating-induced oviposition and to identify the specific receptors involved in this process. Mianserin has commonly been used as a non-specific antagonist of octopamine receptors in insects (Farooqui et al., 2003; Maqueira et al., 2005; Fussnecker et al., 2006; Li et al., 2016). Phentolamine and epinastine have also been reported as specific antagonists for octopaminergic receptors in insects (Evans, 1984; Lange and Orchard, 1986; Degen and Gewecke, 1998; Roeder et al., 1998; Wu et al., 2017). The inhibition of oviposition of mated females by mianserin, phentolamine, and epinastine indicates that octopaminergic receptors are essential in oviposition by *P. xylostella*.

Oviposition is a complex behavior orchestrated by a series of processes. The development and maturation of oocytes occurred at ovaries (Wigglesworth, 1964). Every oocyte is covered by an egg chamber formed by a layer of somatic follicle cells (Spradling, 1993). After the oocytes are matured, follicles start rupturing and allow mature eggs to leave ovaries (Deady and Sun, 2015). Next the eggs are transferred through oviducts by oviductal peristalsis and get fertilized by binding with spermatozoa transferred from spermatheca. Finally the eggs are deposited by forceful constriction of the bursa. (Davey, 1958) Most research has focused on the concept that octopamine and tyramine stimulates ovulation by controlling oviductal muscle contractions (Lange and Orchard, 1986; Nykamp and Lange, 2000; Donini and Lange, 2004; Lee et al., 2009; Lim et al., 2014). Indeed, the expanding of oviduct could help mature eggs to be transferred into it (Hana and Lange, 2017). In locusts, *L. migratoria*, and blood-feeding bugs, *Rhodnius prolixus*, octopamine and tyramine inhibit the contractions of oviducts by increasing intracellular cAMP levels (Lange and Orchard, 1986; Nykamp and Lange, 2000; Donini and Lange, 2004). Chowański et al. (2017) also found that octopamine and tyramine both decrease the frequency of oviducts contraction in a beetle, *Tenebrio molitor*. Since increasing cAMP levels is the main function of activated Octβ-Rs, it is normally thought that the regulation of oviduct contractions was accomplished via Octβ-Rs. In the brown planthopper, *Nilaparvata lugens*, Octβ2R was found to be essential for oviposition and could activate the cAMP levels of the oviducts (Wu et al., 2017). However, phentolamine, a consistently potent antagonist of Octβ-Rs in *P. xylostella* (Huang et al., 2018) and other insect such as rice stem borer, *Chilo suppressalis* (Wu et al., 2012), locusts, *L. migratoria* (Evans, 1984; Lange and Orchard, 1986), and brown planthopper, *N. lugens* (Wu et al., 2017), had a weak inhibitory effect and needed to be administered at a higher concentration than mianserin and epinastine, which are the antagonists of both Octα-Rs and Octβ-Rs. It can be assumed that Octβ-Rs might not be the only and key receptors involved in *P. xylostella* oviposition and can be compensated by Octα-Rs. In *Drosophila*, neither of the mutant females lacking OAMB (α-adrenergic octopamine receptor) or Octβ2R (β-adrenergic octopamine receptor) could ovulate normally. Consistent with our results, two kinds of receptors have both been shown to be expressed in the oviducts, and the expression of OctαR could also rescue the ovulation of an Octβ2R mutant (Lee et al., 2009; Lim et al., 2014). Besides intracellular Ca^2+^ levels, Octα-R can also activate the cAMP/PKA signaling pathway at a relatively lower level than β-adrenergic like receptors (Balfanz et al., 2005). It can be assumed that Octα-R can regulate oviduct contraction by the cAMP/PKA signaling pathway as a supplementary of Octβ2R. Moreover, in the cricket, *Gryllus bimaculatus*, octopamine was also found increasing the spontaneous rhythmic contractions of the lateral oviduct via Ca^2+^ signaling (Tamashiro and Yoshino, 2014). Besides the regulation of oviduct contractions, one of Octα-Rs, OAMB, induces ovarian follicle rupturing and ovulation via intracellular Ca^2+^ increasing and matrix metalloproteinase 2 in *Drosophila* (Deady and Sun, 2015; Deady et al., 2015). Follicle rupturing is a necessary step in releasing eggs from ovaries. Octα-Rs might have distinct functions from relaxing the oviducts in *P. xylostella*. The regulation of octopamine on oviposition in *P. xylostella* might be through more than one receptor and signal pathway. Since little research on the functions of octopaminergic receptors in the reproductive system has been reported, a complete working model of octopamine in reproductive systems is still lacking.

Although tyramine could induce oviposition by *P. xylostella* (Figure 2), yohimbine, the most effective tyramine receptor antagonist in a variety of insects (Saudou et al., 1990; Poels et al., 2002; Huang et al., 2009; Wu et al., 2013) failed to block mating-induced oviposition (Figure 4B). It can be concluded that tyramine receptors are not involved in the oviposition by the diamondback moth. Moreover, tyramine-inducing oviposition could not be inhibited by yohimbine, the potent tyramine receptor antagonist, but could be inhibited by mianserin and epinastine (Figure 4B). It can be hypothesized that tyramine induces oviposition by *P. xylostella* via octopamine receptors instead of tyramine receptors. Consistent with our results, in locusts, *L. migratoria*, and blood-feeding bugs, *R. prolixus*, although tyramine has a significantly higher inhibitory effect on oviduct contraction, yohimbine was unable to diminish this inhibitory effect (Donini and Lange, 2004; Hana and Lange, 2017). Besides regulation on contraction of oviducts, other mechanisms of tyramine-induced ovulation are unclear. More work needs to be done to understand the mechanisms of tyramine-inducing oviposition. As SCH 23390 is the specific and effective antagonist of the D1-like and D2-like dopamine receptors in various invertebrates, such as *Bombyx mori* (Ohta et al., 2009), *Lxodes scapularis* (Ejendal et al., 2012), and *Aedes aegypti* (Meyer et al., 2012), and ketanserin is the selective antagonist of serotonin receptors (Cai et al., 2008; Vleugels et al., 2014), the failure of SCH23390 and ketanserin to block the oviposition by mated *P. xylostella* females indicates that dopaminergic receptors and serotoninergic receptors are not involved in *P. xylostella* oviposition.

In conclusion, both octopamine and tyramine induced oviposition by *P. xylostella* when tyramine had a weaker and assistant effect. Both octopamine and tyramine-inducing oviposition were accomplished via octopaminergic receptors instead of tyraminergic receptors. Octβ-Rs were important but not the only receptors in the regulation of octopamine during *P. xylostella* oviposition. Octopaminergic α and β receptors may both be involved in the regulation of oviposition. Our experiments could contribute to understanding the role of octopaminergic signals on mating-regulated oviposition and provide a new strategy for the control of *P. xylostella*. However, we still have a long way to go to find out the specific mechanisms underlying octopamine and tyramine regulation of oviposition by *P. xylostella*.

## Data Availability Statement

All datasets generated for this study are included in the article/supplementary material.

## Author Contributions

FL, Y-LF, and T-XL designed research and wrote the manuscript. FL performed the research. KL and L-JW provided the assistance. FL analyzed the data.

## Conflict of Interest

The authors declare that the research was conducted in the absence of any commercial or financial relationships that could be construed as a potential conflict of interest.

## References

[B1] Agarwal M. Manuel G. G. Morales-Matos C. Rafael D. Abramson C. I. Giray T. (2011). Dopamine and octopamine influence avoidance learning of honey bees in a place preference assay. *PLos One.* 6:25371. 10.1371/journal.pone.0025371 21980435 PMC3184138

[B2] Akasaka S. Sasaki K. Harano K. Nagao T. (2010). Dopamine enhances locomotor activity for mating in male honeybees (*Apis mellifera* L.). *J. Insect Physiol.* 56 1160–1166. 10.1016/j.jinsphys.2010.03.013 20303974

[B3] Al-Wathiqui N. Dopman E. B. Lewis S. M. (2016). Postmating transcriptional changes in the female reproductive tract of the European corn borer moth. *Insect Mol Biol.* 25 629–645. 10.1111/imb.12249 27329655

[B4] Avila F. W. Sirot L. K. Laflamme B. A. Rubinstein C. D. Wolfner M. F. (2011). Insect seminal fluid proteins: identification and function. *Annu Rev. Entomol.* 56 21–40. 10.1146/annurev-ento-120709-144823 20868282 PMC3925971

[B5] Balfanz S. Strünker T. Frings S. Baumann A. (2005). A family of octapamine receptors that specifically induce cyclic amp production or ca2+ release in *Drosophila melanogaster*. *J. Neurochem* 93 440–451. 10.1111/j.1471-4159.2005.03034.x 15816867

[B6] Blenau W. Balfanz S. Baumann A. (2000). Amtyr1: characterization of a gene from honeybee (*Apis mellifera*) brain encoding a functional tyramine receptor. *J. Neurochem.* 74 900–908. 10.1046/j.1471-4159.2000.0740900.x 10693920

[B7] Blenau W. Baumann A. (2001). Molecular and pharmacological properties of insect biogenic amine receptors: lessons from *Drosophila melanogaster* and *Apis mellifera*. *Arch Insect Biochem Physiol.* 48 13–38. 10.1002/arch.1055 11519073

[B8] Blenau W. Thamm M. (2011). Distribution of serotonin (5-HT) and its receptors in the insect brain with focus on the mushroom bodies. Lessons from *Drosophila melanogas*ter and *Apis mellifera*. *Arthropod Struct Dev.* 40 381–394. 10.1016/j.asd.2011.01.004 21272662

[B9] Blumenthal E. M. (2003). Regulation of chloride permeability by endogenously produced tyramine in the *Drosophila malpighian* tubule. *AJP Cell Physiol.* 284 718–728. 10.1152/ajpcell.00359.2002 12444020

[B10] Boulay R. Hooper-Bui L. M. Woodring J. (2001). Oviposition and oogenesis in virgin fire ant females *Solenopsis invicta* are associated with a high level of dopamine in the brain. *Physiol Entomol.* 26 294–299. 10.1046/j.0307-6962.2001.00250.x

[B11] Brent S. C. Miyasaki K. Vuong C. Miranda B. Steele B. Brent G. K. (2016). Regulatory roles of biogenic amines and juvenile hormone in the reproductive behavior of the western tarnished plant bug (*Lygus hesperus*). *J. Comp Physiol B.* 186 169–179. 10.1007/s00360-015-0953-1 26686231

[B12] Cai M. Y. Li Z. Huang Q. Song G. (2008). {[1-(Arylmethyl) piperidin-4-yl] oxy}-(trifluoromethyl)-pyridines: ketanserin analogues with insect growth regulating activity. *Chem Biodivers.* 5 1844–1855. 10.1002/cbdv.200890172 18816514

[B13] Carmel I. Tram U. Heifetz Y. (2016). Mating induces developmental changes in the insect female reproductive tract. *Curr Opin. Insect Sci.* 13 106–113. 10.1016/j.cois.2016.03.002 27436559

[B14] Chen N. Fan Y. L. Bai Y. Li X. D. Liu T. X. (2016). Cytochrome p450 gene, cyp4g51, modulates hydrocarbon production in the pea aphid. *Acyrthosiphon pisum*. Insect Biochem Mol Biol. 76 84–94. 10.1016/j.ibmb.2016.07.006 27425674

[B15] Chowański S. Spochacz M. Szymczak M. Rosiński G. (2017). Effect of biogenic amines on the contractile activity of visceral muscles in the beetle *Tenebrio molitor. B. Insectol.* 70, 209–220.

[B16] Cohen R. W. Mahoney D. A. Can H. D. (2002). Possible regulation of feeding behavior in cockroach nymphs by the neurotransmitter octopamine. *J. Insect. Behav.* 15, 37–50. 10.1023/A:1014428011892

[B17] Cole S. H. Carney G. E. Mcclung C. A. Willard S. S. Hirsh J. (2005). Two functional but noncomplementing *Drosophila* tyrosine decarboxylase genes. *J. Biol Chem.* 280 14948–14955. 10.1074/jbc.M414197200 15691831

[B18] Dacks A. M. Nickel T. Mitchell B. K. (2003). An examination of serotonin and feeding in the flesh fly *Neobellieria bullata* (*Sarcophagidae*: *Diptera*). *J. Insect Behav.* 16 1–21. 10.1023/A:1022817610378

[B19] Davey K. G. (1958). The migration of spermatozoa in the female of *Rhodnius Prolixus* Stål. *J. Exp Biol.* 35, 694–701.

[B20] Deady L. D. Shen W. Mosure S. A. Spradling A. C. Sun J. (2015). Matrix metalloproteinase 2 is required for ovulation and corpus luteum formation in *Drosophila*. *PLoS Genet.* 11:e1004989. 10.1371/journal.pgen.1004989 25695427 PMC4335033

[B21] Deady L. D. Sun J. (2015). A follicle rupture assay reveals an essential role for follicular adrenergic signaling in *Drosophila* ovulation. *PLoS Genet.* 11:e1005604. 10.1371/journal.pgen.1005604 26473732 PMC4608792

[B22] Degen J. Gewecke M. (1998). Epinastine, a highly specific antagonist of insect neuronal octopamine receptors. *Eur J. Pharmacol.* 349 171–177. 10.1016/S0014-2999(98)00192-7 9671095

[B23] Dierick H. A. Greenspan R. J. (2007). Serotonin and neuropeptide F have opposite modulatory effects on fly aggression. *Nat Genet.* 39 678–682. 10.1038/ng2029 17450142

[B24] Donini A. Lange A. B. (2004). Evidence for a possible neurotransmitter/neuromodulator role of tyramine on the locust oviducts. *J. Insect Physiol.* 50 351–361. 10.1016/j.jinsphys.2004.02.005 15081828

[B25] Draper I. Kurshan P. T. Mcbride E. Jackson F. R. Kopin A. S. (2007). Locomotor activity is regulated by d2-like receptors in *Drosophila*: an anatomic and functional analysis. *Dev Neurobiol.* 67 378–393. 10.1002/dneu.20355 17443795

[B26] Ejendal K. F. K. Meyer J. M. Brust T. F. Avramova L. V. Watts V. J. (2012). Discovery of antagonists of tick dopamine receptors via chemical library screening and comparative pharmacological analyses. *Insect Biochem Mol Biol.* 42 846–853. 10.1016/j.ibmb.2012.07.011 23213654

[B27] Evans P. D. (1984). A modulatory octopaminergic neurone increases cyclic nucleotide levels in locust skeletal muscle. *J. Physiol.* 348 307–324. 10.1113/jphysiol.1984.sp015112 6201609 PMC1199404

[B28] Evans P. D. Maqueira B. (2005). Insect octopamine receptors: a new classification scheme based on studies of cloned *Drosophila* G-protein coupled receptors. *Invertebr Neurosci.* 5 111–118. 10.1007/s10158-005-0001-z 16211376

[B29] Farooqui T. (2012). Review of octopamine in insect nervous system. *Open Access Insect Physiol.* 4 1–17. 10.2147/OAIP.S20911

[B30] Farooqui T. Robinson K. Vaessin H. Smith B. H. (2003). Modulation of early olfactory processing by an octopaminergic reinforcement pathway in the honeybee. *J. Neurosci.* 23 5370–5380. 10.1016/S0016-5107(75)73705-7 12832563 PMC6741157

[B31] Fox L. E. Soll D. R. Wu D.-F. (2006). Coordination and modulation of locomotion pattern generators in *Drosophila* larvae: effects of altered biogenic amine levels by the tyramine beta hydroxlyase mutation. *J. Neurosci.* 26 1486–1498. 10.1523/JNEUROSCI.4749-05.2006 16452672 PMC2673197

[B32] French A. S. Simcock K. L. Rolke D. (2014). The role of serotonin in feeding and gut contractions in the honeybee. *J. Insect Physiol.* 61 8–15. 10.1016/j.jinsphys.2013.12.005 24374107 PMC3969292

[B33] Fussnecker B. L. Smith B. H. Mustard J. A. (2006). Octopamine and tyramine influence the behavioral profile of locomotor activity in the honey bee (*Apis mellifera*). *J. Insect Physiol.* 52 1083–1092. 10.1016/j.jinsphys.2006.07.008 17028016 PMC1712669

[B34] Giebultowicz J. M. Raina A. K. Uebel E. C. Ridgway R. L. (1991). Two-step regulation of sex-pheromone decline in mated gypsy moth females. *Arch. Insect. Biochem. Physiol.* 16, 95–105. 10.1002/arch.940160203

[B35] Gillott C. (2003). Male accessory gland secretions: modulators of female reproductive physiology and behavior. *Annu Rev. Entomol.* 48 163–184. 10.1146/annurev.ento.48.091801.112657 12208817

[B36] Gong Y. J. Wang Z. H. Shi B. C. Kang Z. J. Zhu L. Jin G. H. (2013). Correlation between pesticide resistance and enzyme activity in the diamondback moth. *Plutella xylostella*. J. Insect Sci. 13 1–13. 10.1673/031.013.13501 PMC4014041 24766444

[B37] Guan R. B. Li H. C. Fan Y. J. Hu S. R. Christiaens O. Smagghe G. (2018). A nuclease specific to lepidopteran insects suppresses RNAi. *J. Biol Chem.* 293 2011–2021. 10.1074/jbc.RA117.001553 29500196 PMC5912458

[B38] Hammer M. R. Menzel R. (1998). Multiple sites of associative odor learning as revealed by local brain microinjections of octopamine in honeybees. *Learn Mem.* 5 146–156. 10.1101/lm.5.1.146 10454379 PMC311245

[B39] Hana S. Lange A. B. (2017). Octopamine and tyramine regulate the activity of reproductive visceral muscles in the adult female blood-feeding bug, *Rhodnius prolixus*. *J. Exp Biol.* 220(Pt 10), 1830–1183. 10.1242/jeb.156307 28235907

[B40] Hays S. B. Hays R. L. Mims I. S. (1969). Comparative effects of reserpine and serotonin creatine sulfate on oviposition in the house fly. *J. Econom. Entomol.* 62 663–664. 10.1093/aesa/62.3.663 5363337

[B41] Hirashima A. Yamaji H. Takaki Y. Kuwano E. Eto M. (2007). Effect of tyramine and stress on sex-pheromone production in the pre- and post-mating silkworm moth. *Bombyx mori*. J. Insect Physiol. 53 1242–1249. 10.1016/j.jinsphys.2007.06.018 17681526

[B42] Huang J. Ohta H. Inoue N. Takao H. Kita T. Ozoe F. (2009). Molecular cloning and pharmacological characterization of a *Bombyx mori* tyramine receptor selectively coupled to intracellular calcium mobilization. *Insect Biochem Mol Miol.* 39 842–849. 10.1016/j.ibmb.2009.10.001 19833207

[B43] Huang Q. T. Ma H. H. Deng X. L. Zhu H. Liu J. Zhou Y. (2018). Pharmacological characterization of a β-adrenergic-like octopamine receptor in *Plutella xylostella*. *Arch. Insect Biochem. Physiol.* 98:e21466. 10.1002/arch.21466 29691888

[B44] Kapelnikov A. Rivlin P. K. Hoy R. R. Heifetz Y. (2008). Tissue remodeling: a mating-induced differentiation program for the *Drosophila* oviduct. *BMC Dev. Biol.* 8:114. 10.1186/1471-213X-8-114 19063748 PMC2636784

[B45] Kingan T. G. Bodnar W. M. Raina A. K. Shabanowitz J. Hunt D. F. (1995). The loss of female sex pheromone after mating in the corn earworm moth *Helicoverpa zea*: identification of a male pheromonostatic peptide. *Proc. Nati Acad. Sci. U.S.A.* 92 5082–5086. 10.1073/pnas.92.11.5082 7761452 PMC41852

[B46] Kita T. Hayashi T. Ohtani T. Takao H. Ozoe Y. (2016). Amitraz and its metabolite differentially activate α- and β-adrenergic-like octopamine receptors: amitraz actions on octopamine receptors. *Pest Manag Sci.* 73:e4412 10.1002/ps.4412 27484898

[B47] Kumar S. Chen D. Sehgal A. (2012). Dopamine acts through cryptochrome to promote acute arousal in *Drosophila*. *Gene Dev.* 26 1224–1234. 10.1101/gad.186338.111 22581798 PMC3371410

[B48] Lange A. B. (2004). A neurohormonal role for serotonin in the control of locust oviducts. *Arch. Insect. Biochem. Physiol.* 56, 179–190. 10.1002/arch.20010 15274179

[B49] Lange A. B. (2009). Tyramine: from octopamine precursor to neuroactive chemical in insects. *Gen Comp Endocr.* 162 18–26. 10.1016/j.ygcen.2008.05.021 18588893

[B50] Lange A. B. Orchard I. (1986). Identified octopaminergic neurons modulate contractions of locust visceral muscle via adenosine 3′,5′-monophosphate (*cyclic amp*). *Brain Res.* 363, 340–349. 10.1016/0006-8993(86)91020-6 2417668

[B51] Lee H. G. Rohila S. Han K. A. (2009). The octopamine receptor OAMB mediates ovulation via ca2+/calmodulin-dependent protein kinase ii in the *Drosophila* oviduct epithelium. *PLoS One* 4:e4716. 10.1371/journal.pone.0004716 19262750 PMC2650798

[B52] Li H. M. Jiang H. B. Gui S. H. Liu X. Q. Liu H. Lu X. P. (2016). Characterization of a β-adrenergic-like octopamine receptor in the oriental fruit fly. *Bactrocera dorsalis* (Hendel). Int J. Mol Sci. 17 1577–1591. 10.3390/ijms17101577 PMC5085626 27669213

[B53] Lim J. Sabandal P. R. Fernandez A. Sabandal J. M. Lee H. G. Evans P. (2014). The octopamine receptor OCTβ2R regulates ovulation in *Drosophila melanogaster*. *PLoS One.* 9:e104441 10.1371/journal.pone.0104441 PMC4123956 25099506

[B54] Liu Q. Liu S. Kodama L. Driscoll M. Wu M. (2012). Two dopaminergic neurons signal to the dorsal fan-shaped body to promote wakefulness in *Drosophila*. *Curr. Biol.* 22 2114–2123. 10.1016/j.cub.2012.09.008 23022067 PMC3505250

[B55] Liu T.-X. Liang G.-M. Xu Y.-Y. Haseeb M. Chen W. (2008). “Adoption of integrated management strategies for diamondback moth and other crucifer insect pests in Texas, 321-345,” in *Proceedings of The Fifth International Workshop*, eds Shelton A. M. Collins H. L. Zhang Y. J. Wu Q. J. (Beijing: China Agricultural Science and Technology Press), 2008.

[B56] Long T. F. Murdock L. L. (1983). Stimulation of blowfly feeding behavior by octopaminergic drugs. *Proc. Nati Acad. Sci. U.S.A.* 80 4159–4163. 10.2307/14347 16593335 PMC394220

[B57] Ma Z. Guo W. Guo X. Wang X. Denlinger K. D. L. (2011). Modulation of behavioral phase changes of the *Migratory locust* by the catecholamine metabolic pathway. *Proc. Nati Acad. Sci. U.S.A.* 108 3882–3887. 10.2307/41061030 21325054 PMC3053982

[B58] Ma Z. Guo X. Lei H. Li T. Hao S. Kang L. (2015). Octopamine and tyramine respectively regulate attractive and repulsive behavior in locust phase changes. *Sci. Rep.* 5:8036. 10.1038/srep08036 25623394 PMC5389030

[B59] Maqueira B. Chatwin H. Evans P. D. (2005). Identification and characterization of a novel family of *Drosophila*β-adrenergic-like octopamine G-protein coupled receptors. *J. Neurochem.* 94, 547–560. 10.1111/j.1471-4159.2005.03251.x 15998303

[B60] Mentel T. Duch C. Stypa H. Gerhard W. Müller U. Pflüger H. J. (2003). Central modulatory neurons control fuel selection in flight muscle of migratory locust. *J. Neurosci.* 23 1109–1113. 10.1002/neu.1000 12598597 PMC6742287

[B61] Meyer J. M. Ejendal K. F. K. Avramova L. V. Garland-Kuntz E. E. Hill C. A. (2012). A “genome-to-lead” approach for insecticide discovery: pharmacological characterization and screening of *Aedes aegypti* d1-like dopamine receptors. *PLoS Neglect. Trop. D* 6:e1478 10.1371/journal.pntd.0001478 PMC3265452 22292096

[B62] Mizunami M. Unoki S. Mori Y. Hirashima D. Matsumoto Y. (2009). Roles of octopaminergic and dopaminergic neurons in appetitive and aversive memory recall in an insect. *BMC Biol.* 7:46. 10.1186/1741-7007-7-46 19653886 PMC2729297

[B63] Monastirioti M. (1999). Biogenic amine systems in the fruit fly *Drosophila melanogaster*. *Microsco. Res. Techniq.* 45 106–121. 10.1002/(SICI)1097-0029(19990415)45:2<106::AID-JEMT5>3.0.CO;2-3 10332728

[B64] Monastirioti M. (2003). Distinct octopamine cell population residing in the cns abdominal ganglion controls ovulation in *Drosophila melanogaster*. *Dev. Biol.* 264 0–49. 10.1016/j.ydbio.2003.07.019 14623230

[B65] Nagao T. Tanimura T. (1988). Distribution of biogenic amines in the cricket central nervous system. *Anal. Biochem.* 171 33–40. 10.1016/0003-2697(88)90121-2 3407919

[B66] Nykamp D. A. Lange A. B. (2000). Interaction between octopamine and proctolin on the oviducts of *Locusta migratoria*. *J. Insect Physiol.* 46 809–816. 10.1016/s0022-1910(99)00170-5 10742530

[B67] Ohta H. Tsuchihara K. Mitsumasu K. Yaginuma T. Ozoe Y. Asaoka K. (2009). Comparative pharmacology of two d1-like dopamine receptors cloned from the silkworm *Bombyx mori*. *Insect Biochem. Mol. Biol.* 39 342–347. 10.1016/j.ibmb.2009.01.011 19507304

[B68] Ohtani A. Arai Y. Ozoe F. Ohta H. Narusuye K. Huang J. (2006). Molecular cloning and heterologous expression of an α-adrenergic-like octopamine receptor from the silkworm Bombyx mori. *Insect. Mol. Biol.* 15, 763–772. 10.1111/j.1365-2583.2006.00676.x 17201769

[B69] Poels J. Suner M. M. Needham M. Torfs H. Broeck J. V. (2002). Functional expression of a locust tyramine receptor in murine erythroleukaemia cells. *Insect Mol. Biol.* 10 541–548. 10.1046/j.0962-1075.2001.00292.x 11903623

[B70] Rafaeli A. (2004). Enhanced oviposition in the moth, *Plodia interpunctella*, after treatment with aminergic analogues. *J. Stored Prod Res.* 40 331–339. 10.1016/s0022-474x(03)00026-2

[B71] Rodríguez-Valentín R. López-González I. Jorquera R. Labarca P. Zurita M. Reynaud E. (2006). Oviduct contraction in *Drosophila* is modulated by a neural network that is both, octopaminergic and glutamatergic. *J. Cell Physiol.* 209 183–198. 10.1002/jcp.20722 16826564

[B72] Roeder T. (2005). Tyramine and octopamine: ruling behavior and metabolism. *Annu. Rev. Entomol.* 50 447–477. 10.1146/annurev.ento.50.071803.130404 15355245

[B73] Roeder T. Degen J. Gewecke M. (1998). Epinastine, a highly specific antagonist of insect neuronal octopamine receptors. *Eur. J. Pharmacol.* 349, 171–177. 10.1016/S0014-2999(98)00192-7 9671095

[B74] Sasaki K. Harano K. I. (2007). Potential effects of tyramine on the transition to reproductive workers in honeybees (*Apis mellifera* L.). *Physiol. Entomol.* 32 194–198. 10.1111/j.1365-3032.2007.00566.x

[B75] Sasaki K. Yamasaki K. Nagao T. (2007). Neuro-endocrine correlates of ovarian development and egg-laying behaviors in the primitively eusocial wasp (*Polistes chinensis*). *J. Insect Physiol.* 53 940–949. 10.1016/j.jinsphys.2007.03.006 17451741

[B76] Saudou F. Amlaiky N. Plassat J. L. Borrelli E. Hen R. (1990). Cloning and characterization of a *Drosophila* tyramine receptor. *EMBO J.* 9 3611–3617. 10.1002/j.1460-2075.1990.tb07572.x 2170118 PMC1326448

[B77] Scheiner R. Entler B. V. Barron A. B. Scholl C. Thamm M. (2017). The effects of fat body tyramine level on gustatory responsiveness of honeybees (*Apis mellifera*) differ between behavioral castes. *Front. Syst. Neurosci.* 11:55. 10.3389/fnsys.2017.00055 28848405 PMC5550709

[B78] Sinakevitch I. T. Daskalova S. M. Smith B. H. (2017). The biogenic amine tyramine and its receptor (AmTyr1) in olfactory neuropils in the honey bee (*Apis mellifera*) brain. *Front. Syst. Neurosci.* 11:77. 10.3389/fnsys.2017.00077 29114209 PMC5660842

[B79] Sombati S. Hoyle G. (1984). Generation of specific behaviors in a locust by local release into neuropil of the natural neuromodulator octopamine. *J. Neurobiol.* 15 481–506. 10.1002/neu.480150607 6097645

[B80] Spradling A. C. (1993). “Developmental genetics of oogenesis,” in *The Development of Drosophila melanogaster*, eds Bate M Martinez-Arias A (Cold Spring Harbor, NY: Cold Spring Harbor Laboratory Press), 1–70.

[B81] Tamashiro H. Yoshino M. (2014). Signaling pathway underlying the octopaminergic modulation of myogenic contraction in the cricket lateral oviduct. *J. Insect Physiol.* 71 30–36. 10.1016/j.jinsphys.2014.09.010 25281895

[B82] Tomioka K. Ikeda M. Nagao T. Tamotsu S. (1993). Involvement of serotonin in the circadian rhythm of an insect visual system. *Sci. Nat.* 80 137–139. 10.1007/BF01131019

[B83] Verlinden H. (2018). Dopamine signalling in locusts and other insects. *Insect Biochem. Molec* 97 40–52. 10.1016/j.ibmb.2018.04.005 29680287

[B84] Vleugels R. Lenaerts C. Broeck J. V. Verlinden H. (2014). Signalling properties and pharmacology of a 5-ht7-type serotonin receptor from *Tribolium castaneum*. *Insect Mol. Biol.* 23 1109–1113. 10.1111/imb.12076 24330075

[B85] Wigglesworth V. B. (1964). The hormonal regulation of growth and reproduction in insects. *Adv. Insect physiol.* 2 247–336. 10.1016/S0065-2806(08)60076-4

[B86] Wu S. F. Huang J. Ye G. Y. (2013). Molecular cloning and pharmacological characterisation of a tyramine receptor from the rice stem borer, *Chilo suppressalis* (Walker). *Pest Manag. Sci.* 69, 126–134. 10.1002/ps.3378 23129510

[B87] Wu S. F. Jv X. M. Li J. Xu G. J. Cai X. Y. Gao C. F. (2017). Pharmacological characterisation and functional roles for egg-laying of a β-adrenergic-like octopamine receptor in the brown planthopper. *Nilaparvata lugens*. Insect Biochem. Mol. Biol. 87 55–64. 10.1016/j.ibmb.2017.008 28629966

[B88] Wu S. F. Yao Y. Huang J. Ye G. Y. (2012). Characterization of a β-adrenergic-like octopamine receptor from the rice stem borer (*Chilo suppressalis*). *J. Exp. Biol.* 215, 2646–2652. 10.1242/jeb.068932 22786641

[B89] Yamane T. (2014). Genetic variation in the effect of monoamines on female mating receptivity and oviposition in the adzuki bean beetle, *Callosobruchus chinensis* (*Coleoptera*: *Bruchidae*). *BMC Evol. Biol.* 14:172. 10.1186/s12862-014-0172-5 25098756 PMC4360256

[B90] Yamane T. Miyatake T. (2010). Reduced female mating receptivity and activation of oviposition in two *Callosobruchus* species due to injection of biogenic amines. *J. Insect Physiol.* 56 271–276. 10.1016/j.jinsphys.2009.10.011 19895815

[B91] Zhou C. Rao Y. Rao Y. (2008). A subset of octopaminergic neurons are important for *Drosophila* aggression. *Nat. Neurosci.* 11 1059–1067. 10.1038/nn.2164 19160504

